# Intake of 7,8-Dihydroxyflavone During Juvenile and Adolescent Stages Prevents Onset of Psychosis in Adult Offspring After Maternal Immune Activation

**DOI:** 10.1038/srep36087

**Published:** 2016-11-08

**Authors:** Mei Han, Ji-chun Zhang, Wei Yao, Chun Yang, Tamaki Ishima, Qian Ren, Min Ma, Chao Dong, Xu-Feng Huang, Kenji Hashimoto

**Affiliations:** 1 Division of Clinical Neuroscience, Chiba University Center for Forensic Mental Health, Chiba 260-8670, Japan; 2 School of Medicine, University of Wollongong, and Illawarra Health and Medical Research Institute (IHMRI), New South Wales 2522, Australia

## Abstract

Prenatal infection and subsequent abnormal neurodevelopment of offspring is involved in the etiology of schizophrenia. Brain-derived neurotrophic factor (BDNF) and its high affinity receptor, tropomyosin receptor kinase B (TrkB) signaling plays a key role in the neurodevelopment. Pregnant mice exposed to polyriboinosinic-polyribocytidylic acid [poly(I:C)] causes schizophrenia-like behavioral abnormalities in their offspring at adulthood. Here we found that the juvenile offspring of poly(I:C)-treated mice showed cognitive deficits, as well as reduced BDNF-TrkB signaling in the prefrontal cortex (PFC). Furthermore, the adult offspring of poly(I:C)-treated mice showed cognitive deficits, prepulse inhibition (PPI) deficits, reduced BDNF-TrkB signaling, immunoreactivity of parvalbumin (PV) and peroxisome proliferator-activated receptor γ coactivator 1α (PGC-1α) in the prelimbic (PrL) of medial PFC and CA1 of hippocampus. Supplementation of a TrkB agonist 7,8-dihydroxyflavone (1 mg/mL in drinking water) during juvenile and adolescent stages could prevent these behavioral abnormalities, reduced BDNF-TrkB signaling in PFC and CA1, and immunoreactivity of PV and PGC-1α in the PrL of medial PFC and CA1 in the adult offspring from poly(I:C)-treated mice. These findings suggest that early intervention by a TrkB agonist in subjects with ultra-high risk for psychosis may reduce the risk of subsequent transition to schizophrenia.

Prenatal infection could be implicated in the etiology of schizophrenia^1^,^2^,^3^,^4^. Late adolescence and early adulthood are peak times for the onset of schizophrenia as this period generally exposes substantial neurobiological changes in the brain^5^,^6^,^7^. Cognitive impairment and social disabilities are often present before the onset of psychosis^6^,^8^,^9^,^10^. Thus, there is increasing interest in the potential benefit of early pharmacological intervention in schizophrenia^11^.

Maternal immune activation (MIA) in rodents has a great impact on brain development and behavioral abnormalities in their offspring^3^,^12^,^13^,^14^. The offspring of prenatal rodents exposed to polyriboinosinic-polyribocytidylic acid [poly(I:C)] mimics schizophrenia-like behavioral abnormalities in adulthood^14^,^15^. These deleterious effects in the offspring after MIA can be prevented by treatment with antipsychotics (e.g., clozapine and risperidone) during the juvenile period^16^,^17^. However, early treatments with antipsychotics during juvenile and adolescent stages caused long-term changes in cognition and neurobiology^18^,^19^. Thus, the use of antipsychotics during these stages has detrimental side effects on the neurodevelopment process in humans. Therefore, it is necessary to develop safe drugs for preventing the onset of schizophrenia.

Brain-derived neurotrophic factor (BDNF) and its high-affinity receptor tropomyosin receptor kinase B (TrkB) signaling plays a key role in brain neurodevelopment^20^,^21^,^22^. Decreased serum BDNF levels have been found in first-episode or chronic patients with schizophrenia^23^,^24^. Furthermore, the reduction of expression of BDNF and TrkB receptor has been found in the prefrontal cortex and hippocampus of patients with schizophrenia^25^,^26^. The deficits in hippocampal BDNF expression are also found in placentas and offspring after maternal poly(I:C) exposure during pregnancy in rodents^12^,^27^. These findings all suggest that decreased BDNF-TrkB signaling plays a role in the pathophysiology of schizophrenia. The TrkB agonist 7,8-dihydroxyflavone (7,8-DHF)^28^ has shown neuroprotective and cognitive enhancing effects in animal models^29^,^30^,^31^. Therefore, it is of great interest to examine whether supplementation with 7,8-DHF during juvenile and adolescent stages can prevent the onset of schizophrenia-like behavioral abnormalities at adulthood in MIA offspring.

In the present study, we investigated whether the offspring of mice exposed to poly(I:C) in the prenatal period show abnormal behaviors, BDNF-TrkB signaling, immunoreactivity of parvalbumin (PV) and peroxisome proliferator-activated receptor γ coactivator 1α (PGC-1α) in the brain regions, which are implicated in the pathophysiology of schizophrenia^25^,^26^,^32^,^33^. Furthermore, we examined whether supplementation with 7,8-DHF during juvenile and adolescent stages can prevent abnormal behaviors, decreased BDNF-TrkB signaling, and PV and PGC-1α immunoreactivity in adult MIA offspring.

## Results

### Cognitive Deficits and Decreased BDNF-TrkB Signaling in the Juvenile Offspring of Prenatal Mice Exposed to Poly(I:C)

**Figure 1:**
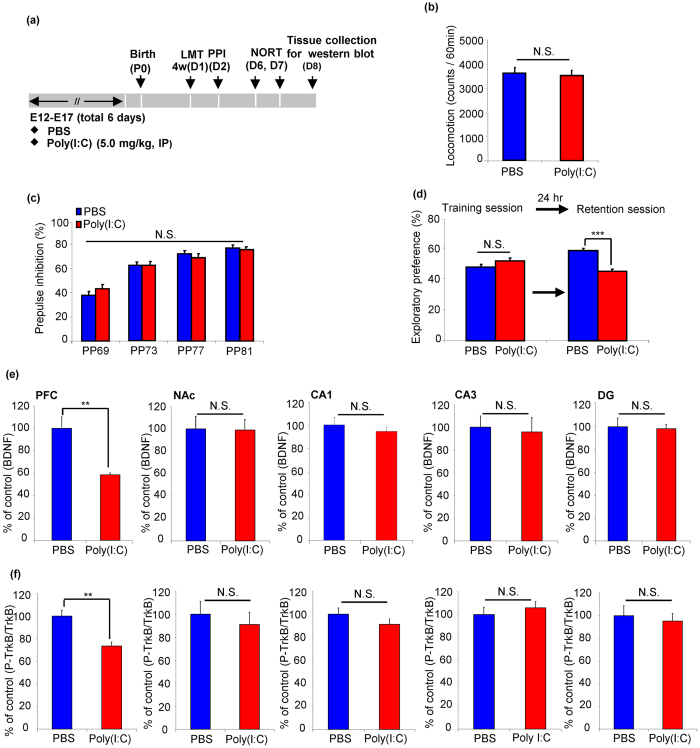
Behaviors and BDNF-TrkB signaling in the juvenile offspring of prenatal mice exposed to poly(I:C). (**a**) Schedule of treatment, behavioral tests and western blot analysis. (**b**) LMT: there were no differences between ploy I:C offspring and controls in locomotor activity. The value is expressed as the mean ± SEM. (n = 13 or 14). (**c**) PPI: there were no differences between poly I:C offspring and controls in the PPI tasks. The value is expressed as the mean ± SEM (n = 16). (**d**) NORT: the exploratory preferences were significantly lower in the poly(I:C) offspring than controls in the retention session, but there was no difference between the two groups in the training session. ***P < 0.001 compared with PBS treated group. The value is expressed as the mean ± SEM (n = 14). (**e**) BDNF: **P < 0.01 compared with PBS treated group. The value is expressed as the mean ± SEM (n = 6). (**f**) p-TrkB/TrkB ratio: **P < 0.01 compared with PBS treated group. The value is expressed as the mean ± SEM (n = 6).

We examined whether offspring from prenatal mice exposed to poly(I:C) could cause abnormal behaviours and BDNF-TrkB signaling in the brain regions at juvenile stage (4–5- weeks old) (Fig. 1a). In the locomotion test (LMT), there was no difference between poly(I:C)-treated offspring and controls (P > 0.05) (Fig. 1b). The MANOVA analysis of PPI data did not reveal significant effects between two groups (Wilks lambda = 0.874, P = 0.44) (Fig. 1c). In the novel object recognition test (NORT), there were no difference between poly(I:C)-treated group and control group in the training session (P > 0.05). However, in the retention session, exploratory preference of poly(I:C)-treated group was significantly lower than that of controls (P < 0.001) (Fig. 1d), suggesting cognitive deficits of MIA offspring at juvenile stage.

BDNF-TrkB signaling plays a key role in many brain functions such as cognition^20^. To examine whether BDNF-TrkB signaling plays a role in the cognitive deficits of juvenile offspring of poly(I:C)-treated group, we measured the levels of BDNF, p-TrkB and TrkB in prefrontal cortex (PFC), nucleus accumbens (NAc) and CA1, CA3, dentate gyrus (DG) of hippocampus in the juvenile offspring of two groups. The levels of BDNF and p-TrkB/TrkB ratio in the PFC of poly(I:C)-treated group were significantly lower than those of controls (Fig. 1e,f). In contrast, the levels of BDNF and p-TrkB/TrkB ratio in the NAc, CA1, CA3 and DG of poly(I:C)-treated group were not different from those of controls (Fig. 1e,f). These results suggest that decreased BDNF-TrkB signaling in the PFC might play a role in the cognitive deficits of juvenile offspring of poly(I:C)-treated group.

### Effect of Supplementation with 7,8-DHF on Behavioral Abnormalities at Adult Offspring of Prenatal Mice Exposed to Poly(I:C)

**Figure 2:**
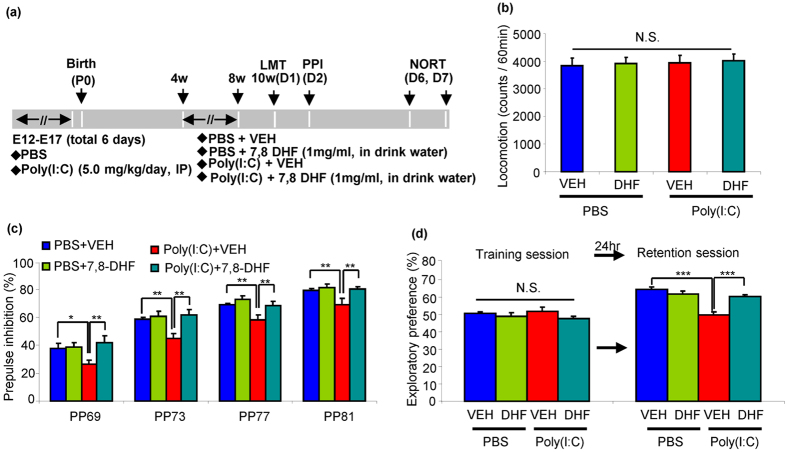
Effect of 7,8-DHF supplementation on behavioural abnormality in the adult offspring of prenatal mice exposed to poly(I:C). (**a**) Schedule of treatment and behavioral tests. (**b**) LMT: there were no significant differences among the four groups in the locomotor activity. The value is expressed as the mean ± SEM (n = 13 or 14). N.S.: not significant. (**c**) PPI: *P < 0.05, **P < 0.01 compared with poly(I:C) + VEH group. The value is expressed as the mean ± SEM (n = 14–16). (**d**) NORT: ***P < 0.001 compared with poly(I:C) + VEH group. The value is expressed as the mean ± SEM (n = 13 or 14).

We examined whether supplementation with 7,8-DHF during juvenile and adolescent period stages could prevent the abnormal behavioural changes at adulthood. The offspring for control and MIA groups were administrated with vehicle or 7,8-DHF (1 mg/mL in drinking water) from 4- to 8- weeks old. Subsequently, normal drinking water was given into all mice for additional 2-weeks (from 8- to 10-weeks old). Behavioural tests were performed at 10–12 weeks olds (Fig. 2a). Two-way ANOVA analysis of LMT data revealed no differences (poly(I:C): F_1,60_ = 0.234, P = 0.630, 7,8-DHF: F_1,60_ = 0.358, P = 0.552, interaction: F_1,60_ = 0.086, P = 0.770) (Fig. 2b). The MANOVA analysis of all PPI data revealed that there were statistically significant effects (Wilks lambda = 0.668, P = 0.031). Post-hoc test indicated that the poly(I:C) + vehicle (VEH) group had significantly lower PPI deficits than phosphate buffered saline (PBS) + VEH or poly(I:C) + 7,8-DHF groups at all dB groups (Fig. 2c). Two-way ANOVA analysis of NORT data in the training session revealed no significant interaction among four groups (poly(I:C): F_1,51_ = 0.000, P = 0.983, 7,8-DHF: F_1,51_ = 3.166, P = 0.081, interaction: F_1,51_ = 0.668, P = 0.418) (Fig. 2d). In the retention session, two-way ANOVA analysis revealed a significant effect among four groups (poly(I:C): F_1,51_ = 30.7, P = 0.000, 7,8-DHF: F_1,51_ = 6.712, P = 0.012, interaction:F_1,51_ = 21.2, P < 0.001) (Fig. 2d). Post-hoc test indicated that exploratory preference of poly(I:C) + VEH group was significantly lower than that of PBS + VEH or poly(I:C) + 7,8-DHF groups (Fig. 2d). These results suggest that adult offspring from pregnant mice exposed to poly(I:C) showed PPI deficits and cognitive deficits at adulthood, and that supplementation with 7,8-DHF from 4- to 8-weeks old could prevent the onset of PPI deficits and cognitive deficits at adulthood after MIA.

### Effect of Supplementation with 7,8-DHF on Decreased BDNF-TrkB Signaling at Adult Offspring after Prenatal Mice Exposed to Poly(I:C)

**Figure 3:**
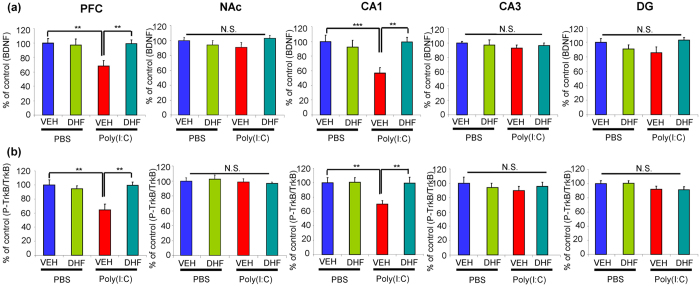
Effect of 7,8-DHF supplementation on decreased BDNF-TrkB signaling in the brain regions of adult offspring of prenatal mice exposed to poly(I:C). (**a**) BDNF: **P < 0.01, ***P < 0.001 compared with poly(I:C) + VEH group. The value is expressed as the mean ± SEM (n = 5 or 6). N.S.: not significant. (**b**) p-TrkB/TrkB: **P < 0.01, ***P < 0.001 compared with poly(I:C) + VEH group. The value is expressed as the mean ± SEM (n = 5 or 6). N.S.: not significant.

In this study, we examined whether supplementation with 7,8-DHF could attenuate decreased BDNF-TrkB signaling in the brain regions of adult offspring after MIA. Two-way ANOVA analysis of BDNF data revealed significant effects (PFC, poly(I:C): F_1,20_ = 4.865, P = 0.039; 7,8-DHF: F_1,20_ = 4.426, P = 0.048; interaction: F_1,20_ = 6.396, P = 0.02), (CA1, poly(I:C): F_1,20_ = 4.841, P = 0.04, 7,8-DHF: F_1,20_ = 4.510, P = 0.046, interaction: F_1,20_ = 9.312, p = 0.006). Post-hoc analysis showed that the levels of BDNF in the PFC and CA1 of the adult offspring from poly(I:C) + VEH group were significantly lower than those of PBS + VEH or poly(I:C) + 7,8-DHF groups in the PFC and CA1 (Fig. 3a). In contrast, there were no differences among the four groups in the other regions including NAc, CA3, and DG.

Two-way analysis of p-TrkB/TrkB ratio data revealed significant effects (PFC, poly(I:C): F_1,20_ = 5.824, P = 0.026; 7,8-DHF: F_1,20_ = 5.305, P = 0.032, interaction: F_1,20_ = 9.683, P = 0.005) (CA1, poly(I:C): F_1,20_ = 5.578, P = 0.028, 7,8-DHF: F_1,20_ = 5.24, P = 0.033, interaction: F_1,20_ = 4.89, P = 0.039). Post-hoc test showed that the p-TrkB/TrkB ratio in the PFC and CA1 of adult offspring from poly(I:C) + VEH group was significantly lower than that of PBS + VEH or poly(I:C) + 7,8-DHF groups (Fig. 3b). In contrast, there were no differences among the four groups in the other regions including NAc, CA3, and DG.

These data suggest that adult offspring from prenatal mice exposed to poly(I:C) showed decreased BNDF-TrkB signaling in the PFC and CA1 of hippocampus, but not NAc, CA3, DG of hippocampus, and that supplementation with 7,8-DHF could normalize decreased BDNF-TrkB signaling in these regions of adult offspring from poly(I:C)-treated group.

### Effect of Supplementation with 7,8-DHF on Decreased PV-Immunoreactivity in the Brain at Adult Offspring after Prenatal Mice Exposed to Poly(I:C)

**Figure 4:**
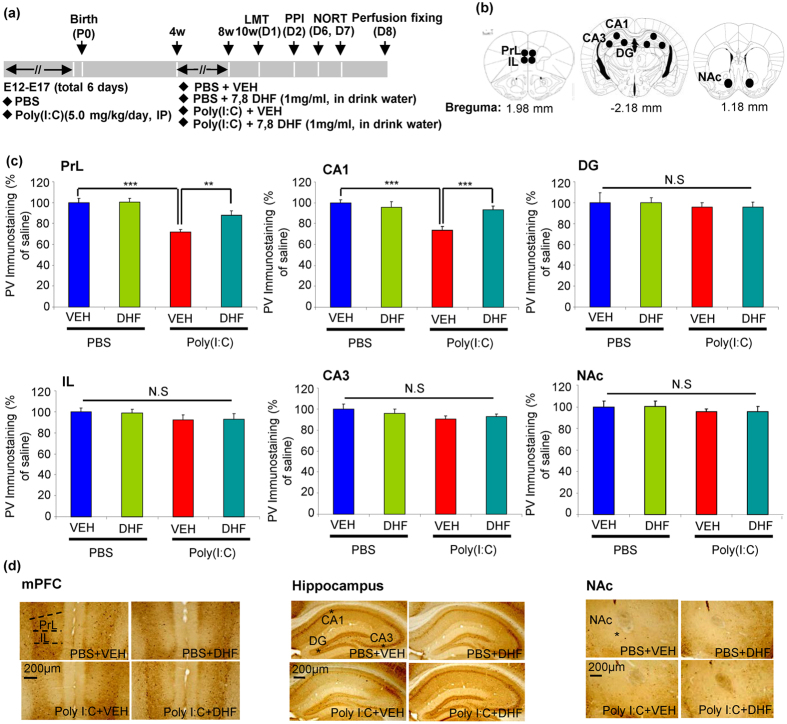
Effect of 7,8-DHF supplementation on loss of PV-immunoreactivity in the brain regions of adult offspring of prenatal mice exposed to poly(I:C). (**a**) Schedule of treatment, behavioural tests and immunohistochemistry. (**b**) Brain regions of the IL and PrL of *mPFC*, NAc, and CA1, CA3, DG of hippocampus are shown. (**c**) PV-immunoreactivity: **P < 0.01, ***P < 0.001 compared with poly(I:C) + VEH group. The value is expressed as the mean ± SEM (n = 6). N.S.: not significant. (**d**) Representative photographs for PV-immunohistochemistry in the brain regions of adult offspring.

Loss of PV-positive cells is known to be associated with cognitive deficits^34^. PV-immunohistochemistry was performed at adulthood (11-weeks old) (Fig. 4a). Two-way ANOVA analysis revealed significant effects (PrL, poly(I:C): F_1,20_ = 29.12, P < 0.001; 7,8-DHF: F_1,20_ = 4.722, P = 0.031; interaction: F_1,20_ = 4.311, P = 0.039) (CA1, poly(I:C): F_1,20_ = 18.203, P < 0.001, 7,8-DHF: F_1,20_ = 4.245, P = 0.041, interaction: F_1,20_ = 11.722, P = 0.001). Post-hoc test showed that PV-immunoreactivity in the PrL of medial PFC (mPFC) and CA1 of hippocampus (not IL, NAc, CA3, and DG) of poly(I:C) + VEH group was significantly lower than that of PBS + VEH or poly(I:C) + 7,8-DHF groups (Fig. 4b–d). These findings suggest that adult offspring from prenatal mice exposed to poly(I:C) showed the loss of PV-immunoreactivity in the PrL of mPFC and CA1, but not NAc, CA3, and DG, and that supplementation with 7,8-DHF could prevent the loss of PV-immunoreactivity in these brain regions after MIA.

### Effect of Supplementation with 7,8-DHF on Decreased PGC-1α Immunoreactivity in the Brain at Adult Offspring after Prenatal Mice Exposed to Poly(I:C)

**Figure 5:**
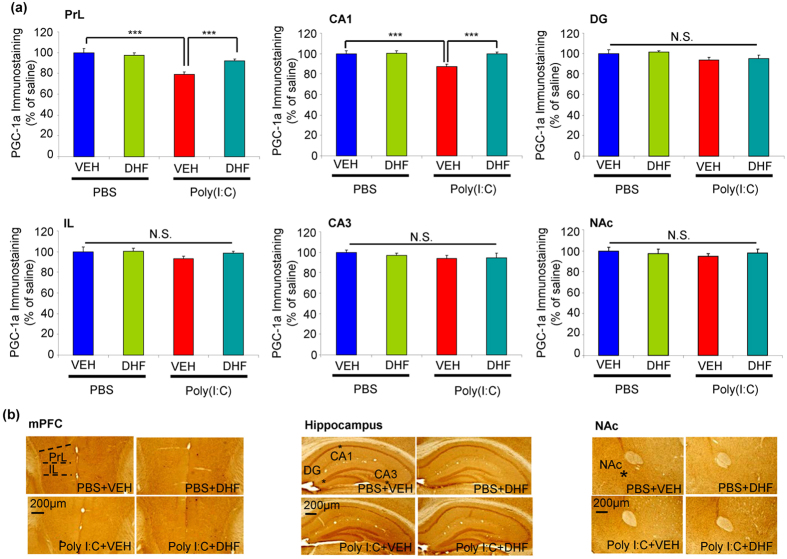
Effect of 7,8-DHF supplementation on loss of PGC-1α immunoreactivity in the brain regions of adult offspring of prenatal mice exposed to poly(I:C). (**a**) PGC-1α immunoreactivity: ***P < 0.001 compared with poly(I:C) + VEH group. The value is expressed as the mean ± SEM (n = 6). N.S.: not significant. (**b**) Representative photographs for PGC-1α-immunohistochemistry in the brain regions of adult offspring.

PGC-1α is a master regulator of metabolism, and is associated with cellular growth, differentiation, and energy metabolism^35^. Furthermore, PGC-1α may regulate the expression of PV within PV-interneurons in the cortex and hippocampus^36^,^37^. We also performed immunohistochemistry of PGC-1α in the brain regions (Fig. 5a,b). Two-way ANOVA analysis revealed significant effects (PrL of mPFC, poly(I:C): F_1,20_ = 24.126, 7,8-DHF: P < 0.001, F_1,20_ = 3.89, P = 0.050, interaction: F_1,20_ = 8.284, P = 0.004)(CA1, poly(I:C): F_1,2 0_ = 7.126, P = 0.008, 7,8-DHF: F_1,20_ = 6.35, P = 0.013, interaction: F_1,20_ = 6.018, P = 0.015). Post-hoc test showed that PGC-1α immunoreactivity in the PrL of mPFC and CA1 (not IL, NAc, CA3, and DG) of poly(I:C) + VEH group was significantly lower than that of PBS + VEH or poly(I:C) + 7,8-DHF groups (Fig. 5a,b). These findings suggest that adult offspring from prenatal mice exposed to poly(I:C) showed loss of PGC-1α immunoreactivity in the PrL of mPFC and CA1, but not NAC, CA3, and DG, and that supplementation with 7,8-DHF could prevent the loss of PGC-1α immunoreactivity in these regions of adult offspring after MIA.

## Discussion

The major findings of the present study are as follows. Supplementation with 7,8-DHF during the juvenile and adolescent stages of offspring of prenatal mice exposed to poly(I:C) led to the prevention of behavioral changes (e.g., cognitive deficits and PPI deficits), decreased BDNF-TrkB signaling in PFC and CA1 of hippocampus, and loss of PV and PGC-1α immunoreactivity in the PrL of mPFC and CA1 of hippocampus at adulthood after MIA. Therefore, it is likely that supplementation with a TrkB agonist such as 7,8-DHF during the prodromal stage has prophylactic effects on the behavioral abnormalities relevant to schizophrenia and related disorders at adulthood.

In this study, we identified the cognitive deficits of offspring from poly(I:C)-treated mice at 4 weeks and 10 weeks of age. Since cognitive deficits are seen in young subjects with ultra-high risk for psychosis^8^,^9^, the juvenile offspring from poly(I:C)-treated mice may be at the prodromal stage for psychosis. Furthermore, we noticed PPI deficits in offspring from poly(I:C)-treated mice at adulthood, but not in those at the juvenile stage. Because PPI deficits in neural circuits might cause some symptoms of schizophrenia^38^,^39^, PPI deficits of adult offspring from poly(I:C)-treated mice mimic PPI deficits in patients with schizophrenia^40^. Our findings are similar to the behavioral abnormalities relevant to human psychiatric disorders including schizophrenia^6^,^10^,^25^. We found that decreased BDNF-TrkB signaling in the PFC and CA1 of the hippocampus may play a role in the cognitive deficits observed in offspring from poly(I:C)-treated mice and that supplementation of 7,8-DHF at 4–8 weeks of age (similar to juvenile and adolescent stages in human) in poly(I:C) offspring could treat or prevent cognitive deficits and PPI deficits at adulthood after MIA. In contrast, we found an increase expression of *Bdnf* gene in the PFC of offspring after poly(I:C) injection (Supplemental Fig. 1). It is likely that increase in *Bdnf* gene expression in the PFC may be compensatory response to decreased expression of BDNF protein in the PFC of offspring from poly(I:C)-treated mice. Prenatal infection may cause neurodevelopmental disorders in their offspring^41^, and downregulation of BDNF-TrkB signaling may be involved in this abnormal brain neurodevelopment^21^,^42^,^43^. We have reported that 7,8-DHF could attenuate behavioral abnormalities (e.g., hyperlocomotion, PPI deficits, and behavioral sensitization) and dopaminergic neurotoxicity after methamphetamine administration^44^,^45^. These results suggest that early treatment with a TrkB agonist during juvenile and adolescent stages may have prophylactic and therapeutic effects on behavioral abnormalities in several psychiatric disorders, including schizophrenia and substance abuse.

Adolescence is a critical period of neurodevelopment, and is also more vulnerable to psychiatric disorders^5^,^7^. The study provides support for the deleterious effects of early brain insult on adult behaviors and brain neurodevelopment abnormalities^46^. Therefore, although adolescence is the peak time for the onset of a number of psychiatric disorders, cognitive deficits at an early age such as the juvenile stage may present prodromal symptoms for later onset of psychiatric disorders. Supplementation with 7,8-DHF in young subjects at ultra-high risk for psychosis may play an important role in preventing the onset of psychosis.

We observed loss of PV immunoreactivity in the PrL, but not IL (infralimbic), of mPFC and CA1 of hippocampus at adulthood after MIA, which is consistent with the findings of other rodent studies^47^,^48^. Loss of PV-positive cells in the PFC might contribute to the pathogenesis of schizophrenia^33^,^34^,^49^. Since TrkB is predominantly expressed by PV-containing neurons in the PFC and hippocampus, decreased BDNF-TrkB signaling in PV-positive interneurons of PrL of mPFC and CA1 of hippocampus may play an important role in modulating behavioral abnormalities in the offspring after MIA.

Further, we found loss of PGC-1α immunoreactivity in the PrL, but not IL, of mPFC and CA1 of hippocampus in the adult offspring after MIA. The mPFC is a heterogeneous cortical structure composed of several nuclei, including PrL and IL cortices^50^. PrL is mainly involved in cognitive function, whereas IL appears to represent a visceromotor center homologous in primates^50^. PrL and IL play an important role in cognitive processes and visceromotor functions, respectively. Therefore, loss of PV immunoreactivity in the PrL, but not IL, of mPFC of adult offspring might be associated with cognitive deficits in MIA offspring. A recent study demonstrated a reduction in PGC-1α-dependent transcripts (e.g., PV, synaptotagmin, and complexin 1) in the anterior cingulate cortex from schizophrenia^32^. Furthermore, a recent study reported an abnormal alteration in the PGC-1α mRNA expression in the PFC from patients with schizophrenia^51^. Taken together, it is likely that reductions in the PV and PGC-1α immunoreactivity in the PrL of mPFC and CA1 of offspring from poly(I:C)-treated mice may be associated with reduced BDNF-TrkB signaling in these regions, supporting the results of a previous study showing BDNF expression through PGC-1α^52^. However, precise studies reporting the underlying BDNF-TrkB signaling through PGC-1α in neurodevelopment are needed.

In conclusion, supplementation of 7,8-DHF during juvenile and adolescent stages could prevent the onset of behavioral abnormalities and loss of PV and PGC-1α immunoreactivity in the PrL of mPFC and CA1 in the offspring after MIA. Therefore, supplementation of a TrkB agonist in young subjects at ultra-high risk for psychosis may prevent conversion to psychosis.

## Methods

### Animals

Female ddY mice (pregnant 5 days) were purchased from SLC Japan (Hamamatsu, Shizuoka, Japan). The first day after copulation was defined as embryonal day 0 (E0) of the pregnancy. Every 6 consecutive days from E12 to E17, the pregnant mice were injected intraperitoneally (IP) with poly(I:C) (5.0 mg/kg) dissolved in 0.2 ml 1% PBS per 20 g body weight or an equivalent volume of PBS, as previously reported^14^. The offspring were separated from their mothers after 3 weeks, and male mice were used for the experiment and caged separately in groups of three to five. The mice were treated with VEH or 7,8-DHF (1 mg/mL) in drinking water for consecutive from 4 to 8-weeks old. Subsequently, normal water was given into all mice from 8 to 10-weeks old. The mice were housed in clear polycarbonate cages (22.5 × 33.8 × 14.0 cm), under a controlled 12/12 hour light-dark cycle (lights on from 07:00 am to 07:00 pm), with room temperature at 23 ± 1 °C and humidity at 55 ± 5%. The mice were given free access to water and food pellets. All experiments were carried out in accordance with the Guide for Animal Experimentation of Chiba University. The protocol was approved by the Chiba University Institutional Animal Care and Use Committee.

### Drugs

Poly(I:C) was purchased from CALBIOCHEM (San Diego, CA). 7,8-DHF was purchased from Tokyo Chemical Industry Co., Ltd. (Tokyo, Japan), and were dissolved in phosphate buffered saline containing 17% dimethyl sulfoxide (DMSO) to generate a stock solution at 100 mg/mL concentration. The stock solution (1 ml) was added to 100 ml of Hydropac water that contained 1% sucrose (pH = 7.4), and subsequently was given to mice as drinking water (1 mg/mL of 7,8-DHF). The dose of 7,8-DHF was selected as previously reported^53^. The VEH contained the same concentration of sucrose and DMSO. The drinking water that contained either 7,8-DHF or VEH was replaced every 2 days. Other drugs were purchased from commercial sources.

### Behavioral tests

#### Locomotion (LMT)

The mice were measured locomotor activity using an animal movement analysis system (SCANET MV-40; Melquest, Toyama, Japan) as reported previously^54^. The locomotion activity was measured 60 min.

#### Prepulse inhibition (PPI)

The mice were tested for their acoustic startle responses in a startle chamber (SR-LAB, San Diego Instruments, San Diego, California, USA) using standard methods described previously^54^. The mice were subjected to one of six trial types: (1) pulse alone, 40-millisecond broadband burst; pulse preceded 100 milliseconds by a 20-millisecond prepulse that was (2) 4 dB (PP69), (3) 8 dB (PP73), (4) 12 dB (PP77) and (5) 16 dB (PP81) over background (65 dB); and (6) background only (no stimulus). The amount of PPI was expressed as the percentage decrease in the amplitude of the startle response caused by presentation of the prepulse (%PPI).

#### Novel object recognition test (NORT)

To assess the cognitive function, the mice were examined by NORT as previously reported^55^. Each mouse habituated in the open field 10 minutes every time for 3 days before the training session. During the training session, two novel objects (differing in shape and colour but of similar size) were placed into the box 35.5 cm apart (symmetrically), and each mouse was allowed to explore freely in the open field for 10 minutes. The mice were considered to be exploring the object when the head of the mouse was touching or standing on the object. The time that mice spent exploring each object was recorded. After training, mice were immediately returned to their home cages, and the box and objects were cleaned with 75% ethanol, to avoid any possible instinctive odorant cues. Retention tests were carried out at the same box 24 hour after the training session, and one of the familiar objects during training was replaced by a novel object. The mice were then allowed to explore freely for 5 minutes, and the time spent exploring each object was recorded. Throughout the experiments, the objects were counter-balanced, in terms of their physical complexity and emotional neutrality. A preference index, a ratio of the amount of time spent exploring either of the two objects (training session) or the novel one (retention session) over the total time spent exploring both objects, was used to measure recognition memory.

### Western blot analysis

The brain samples of prefrontal cortex (PFC), CA1, CA3 and dentate gyrus (DG) of the hippocampus, and nucleus accumbens (NAc) were prepared and Western blot analysis was performed as described previously^56^,^57^,^58^. Basically, tissue samples were homogenized in Laemmli lysis buffer. Aliquots (10 μg) of protein were measured using the DC protein assay kit (Bio-Rad, Hercules, CA, USA), and incubated for 5 min at 95 °C, with an equal volume of 125 mM Tris/HCl, pH 6.8, 20% glycerol, 0.1% bromophenol blue, 10% β-mercaptoethanol, 4% sodium dodecyl sulfate, and subjected to sodium dodecyl sulfate polyacrylamide gel electrophoresis, using 10% mini-gels (Mini-PROTEAN^®^ TGX™ Precast Gel; Bio-Rad, CA, USA). Proteins were transferred onto polyvinylidenedifluoride (PVDF) membranes using a Trans Blot Mini Cell (Bio-Rad). For immunodetection, the blots were blocked with 2% bovine serum albumin (BSA) in TBST (TBS + 0.1% Tween-20) for 1 hour at room temperature (RT), and kept with primary antibodies overnight at 4 °C. The following primary antibodies were used: BDNF (1:1000, H-117, Santa Cruz Biotechnology, Inc., CA, USA), phosphorylated-TrkB (Tyr 706) (1:200, Santa Cruz Biotechnology), TrkB (80E3) (1:1000, Cell Signalling Technology, Danvers, MA, USA) and β-actin (1:10000, Sigma-Aldrich). The next day, blots were washed three times in TBST and incubated with horseradish peroxidase conjugated anti-rabbit antibody or anti-mouse antibody for 1 hour at room temperature. After final three washes with TBST, bands were detected using enhanced chemiluminescence (ECL) plus the Western Blotting Detection system (GE Healthcare Bioscience, Tokyo, Japan). Images were captured with a Fuji LAS3000-mini imaging system (Fujifilm, Tokyo, Japan) and immunoreactive bands were quantified.

### Immunohistochemistry

Immunohistochemistry on the mouse brain sections was performed as the reported previously^57^,^59^,^60^. Mice were deeply anesthetized with sodium pentobarbital and perfused transcardially with 10 ml of isotonic saline, followed by 40 ml of ice-cold, 4% paraformaldehyde in a 0.1 M phosphate buffer (pH 7.4). Brains were removed from the skulls and postfixed overnight at 4 °C in the same fixative. For the immunohistochemical analysis, 50 μm-thick serial, coronal sections of brain tissue were cut in ice-cold, 0.01 M phosphate buffered saline (pH 7.5) using a vibrating blade microtome (VT1000s, Leica Microsystems AG, Wetzlar, Germany). Free-floating sections were treated with 0.3% H_2_O_2_ in 50 mM Tris-HCL saline (TBS) for 30 min and were blocked in TBS containing 0.2% Triton X-100 (TBST) and 1.5% normal goat serum for 1 h at room temperature. The samples were then incubated for 24 h at 4 °C with rabbit polyclonal anti-PV antibody (1:2,500, Swant, Bellinzona, Switzerland) or rabbit polyclonal anti-PGC-1α antibody (1:500, Abcam, Cambridge, MA, USA). The sections were washed three times in TBS and then processed using the avidin-biotin-peroxidase method (Vectastain Elite ABC, Vector Laboratories, Inc., Burlingame, CA, USA). Sections were incubated for 3 min in a solution of 0.25 mg/mL diaminobenzidine containing 0.01% H_2_O_2_. Then, sections were mounted on gelatinized slides, dehydrated, cleared, and coverslipped under Permount^®^ (Fisher Scientific, Fair Lawn, NJ, USA). The sections were imaged, and the staining intensity of PV or PGC-1α immunoreactivity in the infralimbic (IL) and prelimbic (PrL) of the medial prefrontal cortex (*mPFC*), nucleus accumbens (NAc), and hippocampus (CA1, CA3, DG) regions were analyzed using a light micro-scope equipped with a CCD camera (Olymups IX70, Tokyo, Japan) and the SCION IMAGE software package. Images of sections within mPFC (IL, PrL), NAc and hippocampal (CA1, CA3, DG) regions were captured using a 100× objective with a Keyence BZ-9000 Generation II microscope (Osaka, Japan).

### Statistical Analysis

The data are expressed as the mean ± standard error of the mean (SEM). Analysis was performed using PASW Statistics 20 (formerly SPSS Statistics; SPSS, Tokyo, Japan). For the offspring at 4 week study, the locomotion, NORT and western blot were analysed between the control and poly(I:C) groups by using a Student t-test. The PPI data were analysed by multivariate analysis of variance (MANOVA), followed by Student t-test. At 10 weeks of study all data collected from the offspring including the locomotion, NORT, western blot and immunohistochemistry results, were analysed by two-way ANOVA, followed by post hoc Fisher’s least significant difference (LSD) test. The PPI data was analysed by MANOVA, followed by a post hoc Fisher’s LSD test. Statistical significance was set at P < 0.05.

## Additional Information

**How to cite this article**: Han, M. *et al*. Intake of 7,8-Dihydroxyflavone During Juvenile and Adolescent Stages Prevents Onset of Psychosis in Adult Offspring After Maternal Immune Activation. *Sci. Rep.* **6**, 36087; doi: 10.1038/srep36087 (2016).

**Publisher’s note**: Springer Nature remains neutral with regard to jurisdictional claims in published maps and institutional affiliations.

## Supplementary Material

Supplementary Information
