# Supplementary material for: Intake of 7,8-Dihydroxyflavone During Juvenile and Adolescent Stages Prevents Onset of Psychosis in Adult Offspring After Maternal Immune Activation
Source: Sci Rep. 2016 Nov 8;6:36087. doi: 10.1038/srep36087 (PMC5099694; doi:10.1038/srep36087)
Supplement: Supplementary Information [file srep36087-s1.pdf]

Intake of 7,8-Dihydroxyflavone During Juvenile and Adolescent Stages Prevents Onset of Psychosis in Adult Offspring After Maternal Immune Activation

Mei Han[1,2], Ji-chun Zhang[1], Wei Yao[1], Chun Yang[1], Tamaki Ishima[1], Qian Ren[1], Min Ma[1], Chao Dong[1], Xu-Feng Huang[2], and Kenji Hashimoto*[,1]

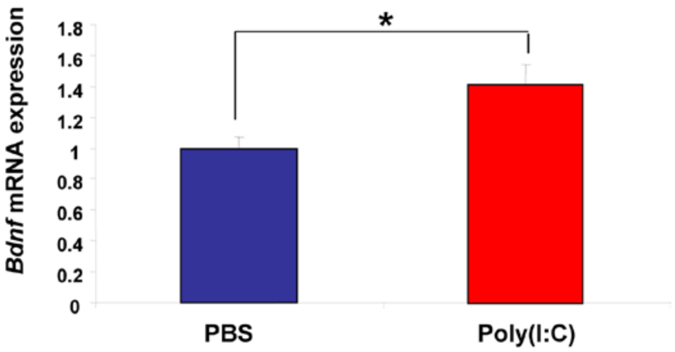

Supplemental Figure S1.

Expression of *Bdnf* mRNA in the PFC of offspring from control (n =10) and poly(I:C)-treated mice (n=14). The data show the mean +/- S.E.M. *P <0.05 (Student t-test)
